# Supplementary figures and images for: Gut-derived peptidoglycan remotely inhibits bacteria dependent activation of SREBP by Drosophila adipocytes
Source: PLoS Genet. 2022 Mar 4;18(3):e1010098. doi: 10.1371/journal.pgen.1010098 (PMC8926189; doi:10.1371/journal.pgen.1010098)

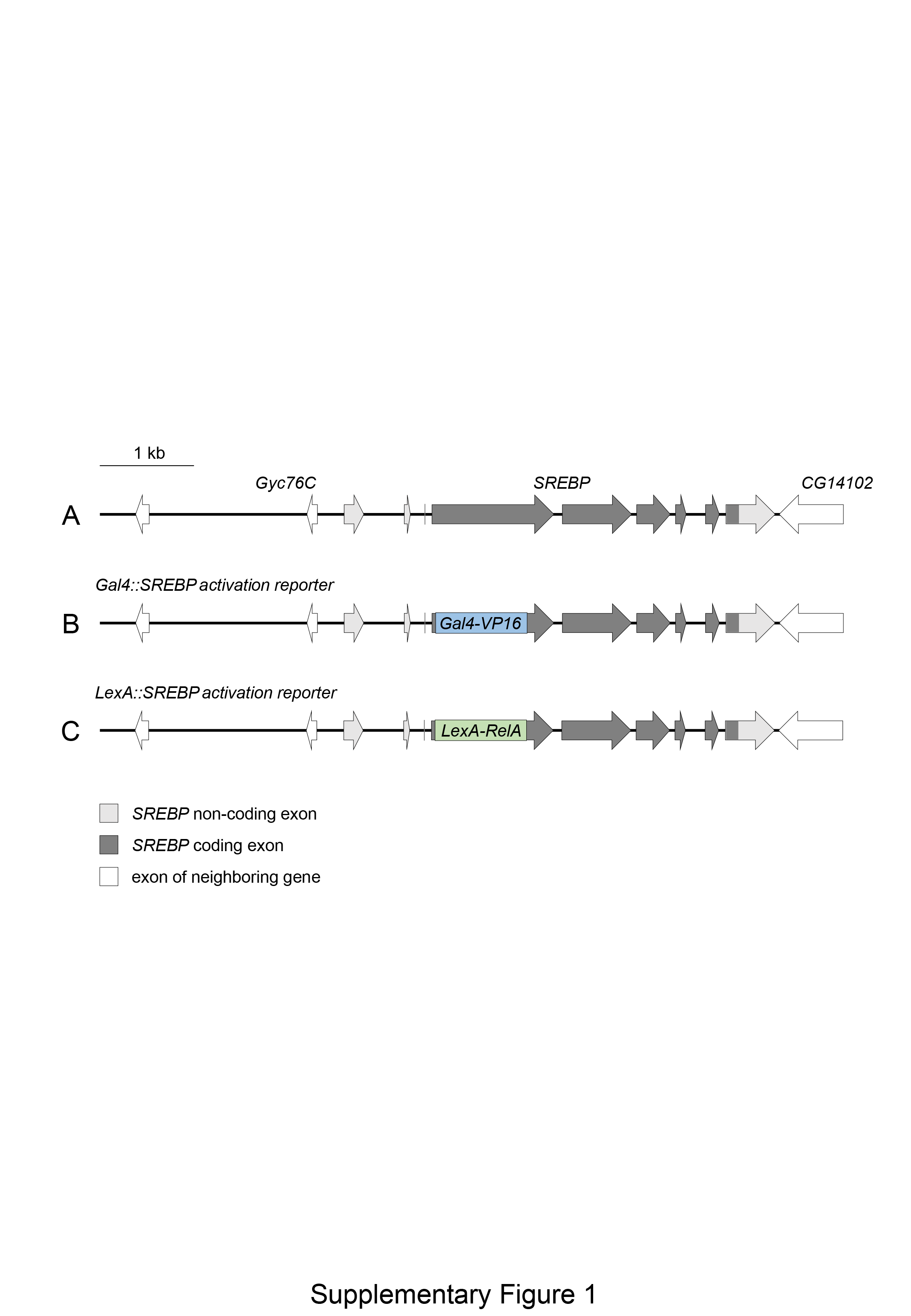

Supplement: S1 Fig — 1, Schematic drawing of the SREBP genomic locus. 2, In the Gal4::SREBP transgene, the transcription factor domain-encoding sequence was replaced by a Gal4::VP16-encoding sequence to report SREBP activation. 3, In the LexA::SREBP transgene the transcription factor domain-encoding sequence was replaced by a LexA::RelA-encoding sequence to report SREBP activation. (TIF) [file pgen.1010098.s001.tif]

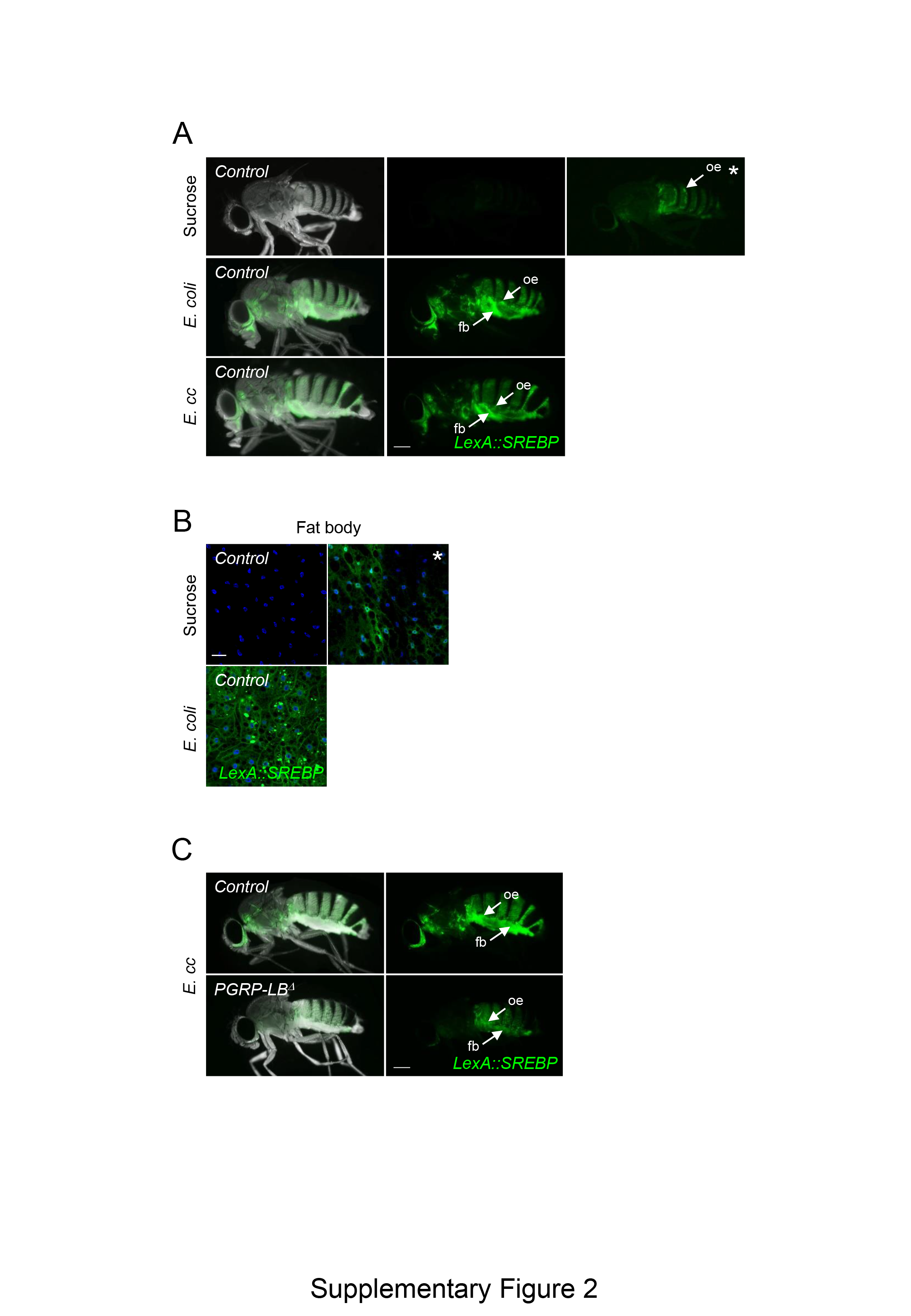

Supplement: S2 Fig — (A) Pictures of adult flies fed 2 days with sucrose, or a mixture of sucrose + E. coli or E. cc, showing LexA::SREBP activation (green). Flies fed with sucrose show activation of LexA::SREBP in oenocytes, noticeable after a longer exposure time (panel with asterisk). Both E. coli and E. cc feeding promotes activation of LexA::SREBP in fat bodies. (B) Confocal images of fat body from flies fed 2 days with sucrose or with E. coli and showing LexA::SREBP activation (green). Flies fed on sucrose show feeble activation of LexA::SREBP in adipocytes, noticeable after increasing the gain during image acquisition (panel with asterisk). E. coli feeding, however, promotes strong activation of LexA::SREBP in adipocytes. (C) Pictures of adult flies, control or PGRP-LBΔ mutant, fed 2 days with sucrose + E. cc, showing LexA::SREBP activation (green). Ingestion of E. cc triggers activation of LexA::SREBP in fat body from control flies, but not from PGRP-LBΔ mutant’s flies. Flies of the following genotypes were used: w1118/w1118, LexA::SREBP, 13XLexAop2-6XGFP/+ (Control in A, B and C), and w1118/w1118, LexA::SREBP, 13XLexAop2-6XGFP/+; PGRP-LBΔ/PGRP-LBΔ (PGRP-LBΔ in C). Scale bar is 0,25 mm (A and C) and 20 μm (B). (TIF) [file pgen.1010098.s002.tif]

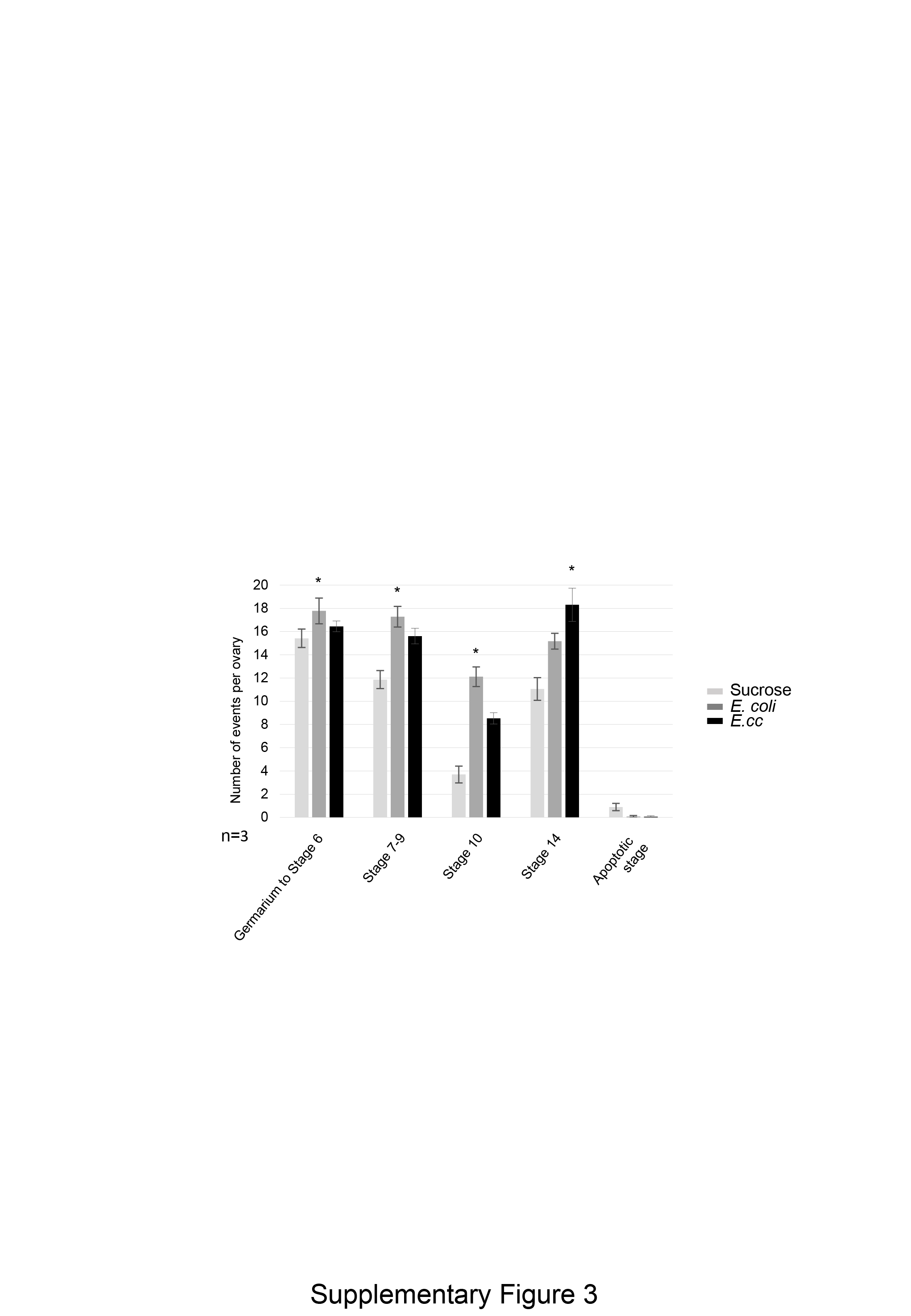

Supplement: S3 Fig — Quantification of the different stages of oocytes observed in female’s ovary, after feeding 24h on sucrose, or on a mixture of sucrose + E. coli or E. cc. Apoptotic events were quantified as oocytes with compact and dense nurse cell nuclei, using DAPI staining. Histograms correspond to the mean value ± SD of three experiments (n = 3). For each oocyte stage, sucrose values were used as reference for statistical analysis. *p<0.05; Kruskal-Wallis test. Flies of the following genotypes were used: w1118/w1118; LexA::SREBP, 13XLexAop2-6XGFP/+. (TIF) [file pgen.1010098.s003.tif]

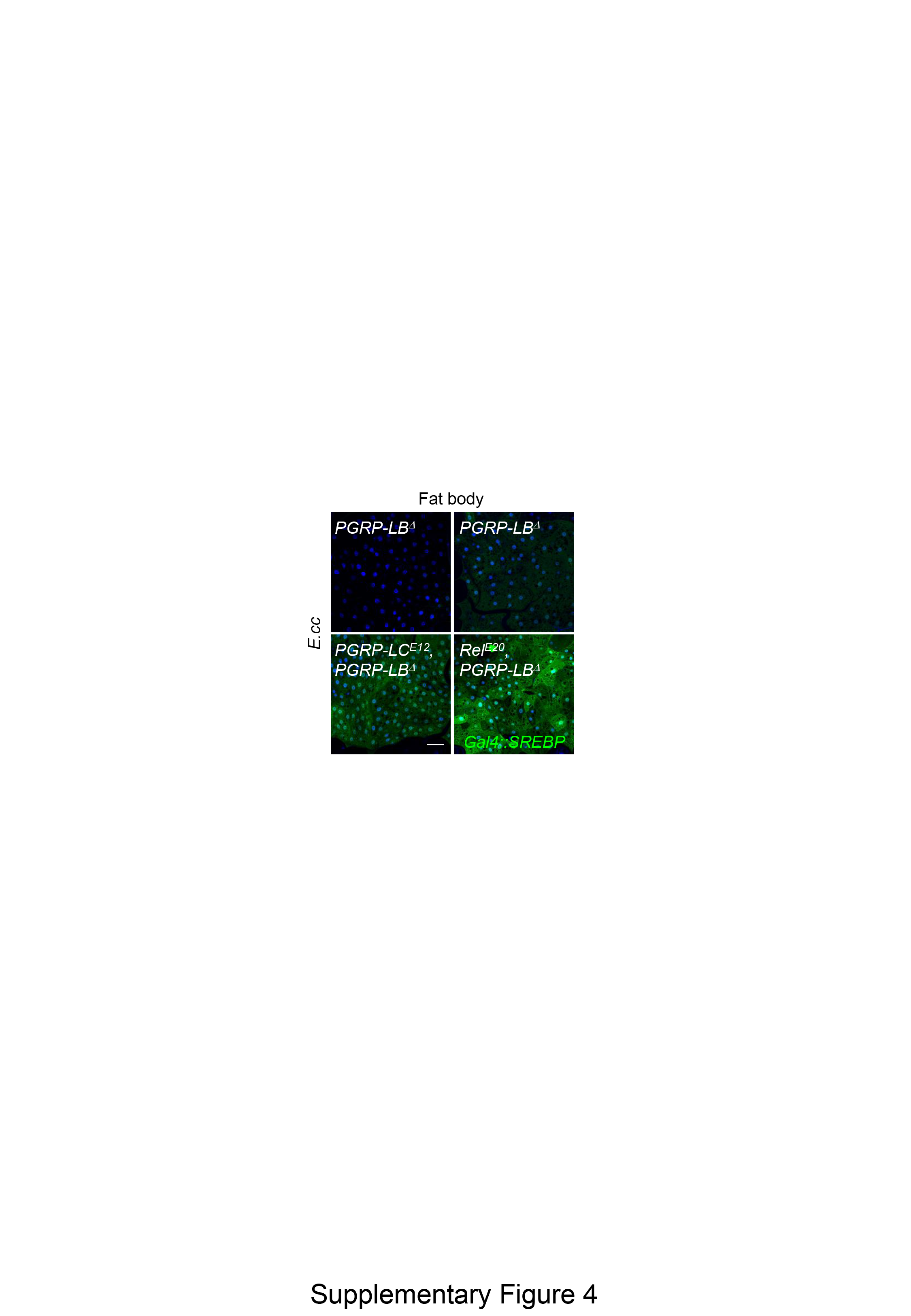

Supplement: S4 Fig — Confocal images of fat body from flies fed 2 days with a mixture of sucrose + E. cc. Double mutant PGRP-LCE12, PGRP-LBΔ or RelE20, PGRP-LBΔ flies activate Gal4::SREBP in adipocytes from flies fed 2 days with E. cc, while PGRP-LBΔ mutants do not. Flies of the following genotypes were used: w1118/w1118; Gal4::SREBP, UAS-2XEGFP/+; PGRP-LCE12, PGRP-LBΔ / PGRP-LBΔ (Top left panel, PGRP-LBΔ) or w1118/w1118; Gal4::SREBP, UAS-2XEGFP/+; PGRP-LCE12, PGRP-LBΔ / PGRP-LCE12, PGRP-LBΔ (PGRP-LCE12, PGRP-LBΔ) or w1118/w1118; Gal4::SREBP, UAS-2XEGFP/+; RelE20, PGRP-LBΔ / PGRP-LBΔ (Top right panel, PGRP-LBΔ) or w1118/w1118; Gal4::SREBP, UAS-2XEGFP/+; RelE20, PGRP-LBΔ / RelE20, PGRP-LBΔ (PGRP-LCE12, PGRP-LBΔ). Scale bar is 20 μm. (TIF) [file pgen.1010098.s004.tif]

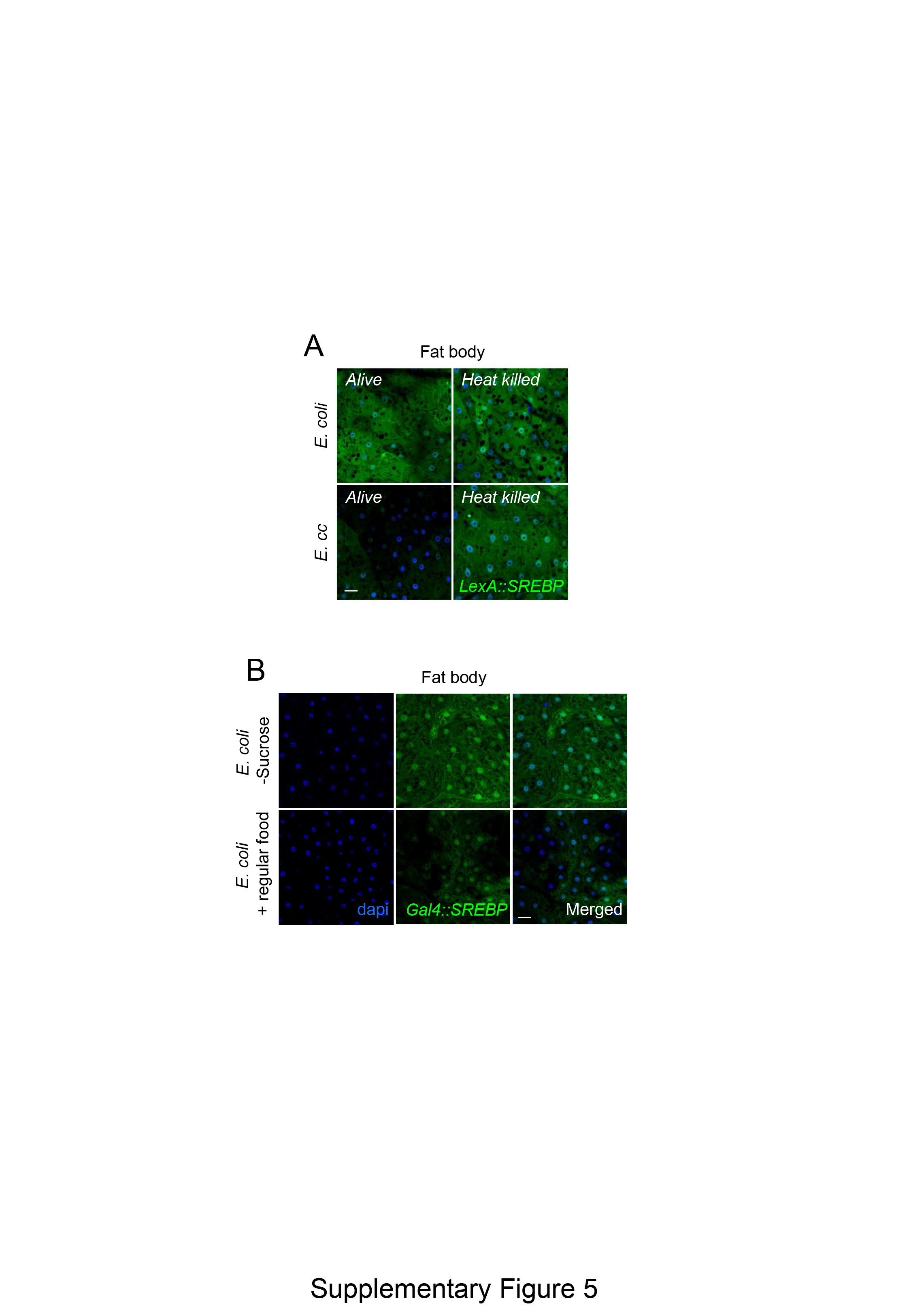

Supplement: S5 Fig — (A) Confocal images of fat body from female flies fed 2 days with a mixture of sucrose + alive or heat killed bacteria (E. coli or E. cc), showing LexA::SREBP activation (green). Both heat killed bacteria are efficiently activating SREBP in adipocytes. (B) Confocal images of fat body from female flies fed 2 days with E. coli without sucrose, or with E. coli dropped on regular food, showing Gal4::SREBP activation (green). Absence of sucrose does not impact the strong activation of SREBP by E. coli, while presence of regular food diminishes it. Flies of the following genotypes were used: w1118/w1118; LexA::SREBP, 13XLexAop2-6XGFP/+ (A) and w1118/w1118; Gal4::SREBP, UAS-2XEGFP/+ (B). Scale bar is 20 μm. (TIF) [file pgen.1010098.s005.tif]

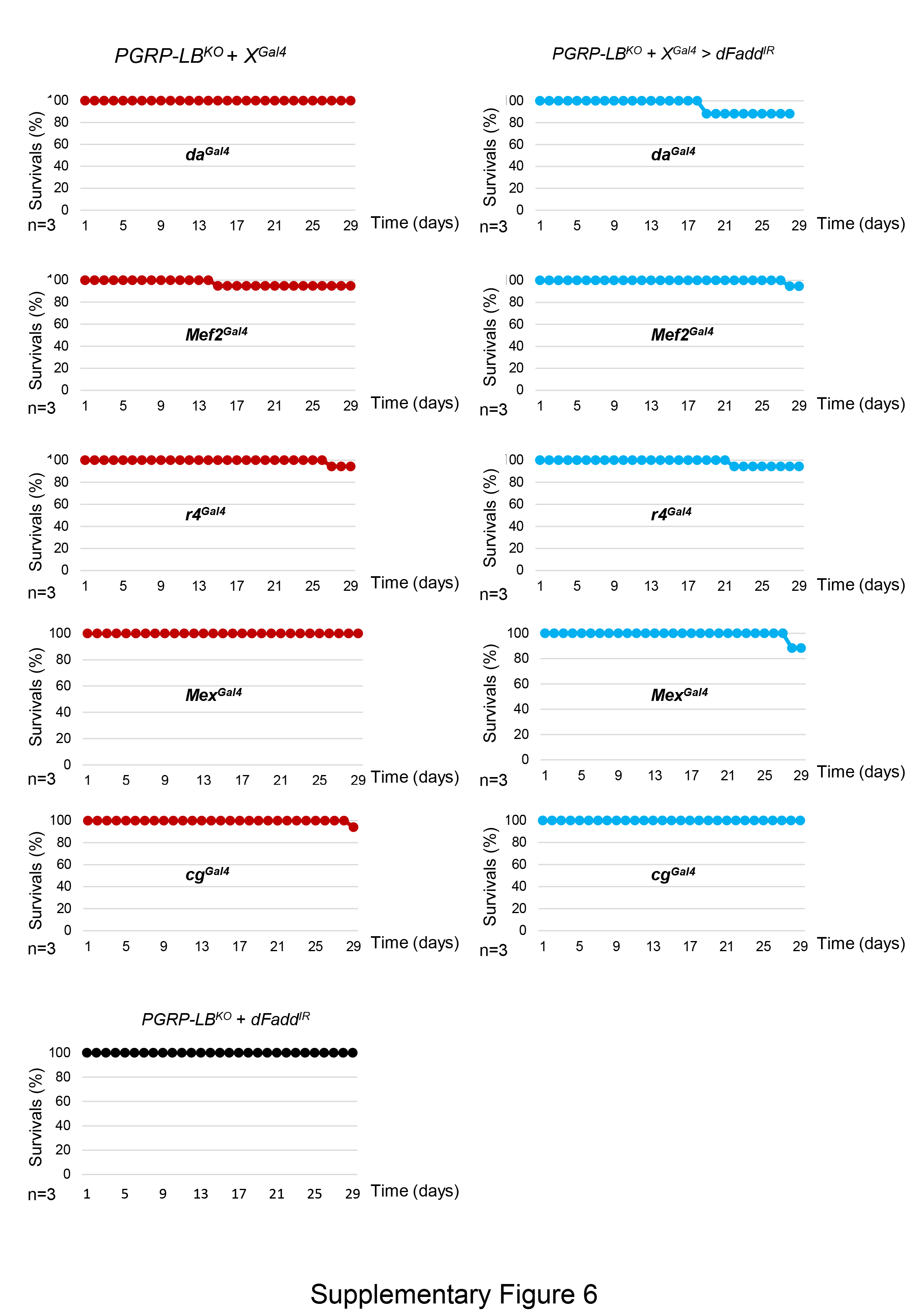

Supplement: S6 Fig — Survival analysis of PGRP-LBΔ mutant flies fed with sucrose. The expression of UAS-dFaddIR ubiquitously using daGal4, or in adipocytes, using either cgGal4 or r4Gal4, or in enterocytes using MexGal4 or in muscle, using Mef2Gal4 have no significant impact on flies’ lifespan, compared to the corresponding control flies. Flies of the following genotypes were used: w1118/w1118;; PGRP-LBΔ, daGal4 or PGRP-LBΔ, Mef2Gal4 or PGRP-LBΔ, r4Gal4/PGRP-LBΔ, UAS-dFaddIR. (TIFF) [file pgen.1010098.s006.tiff]

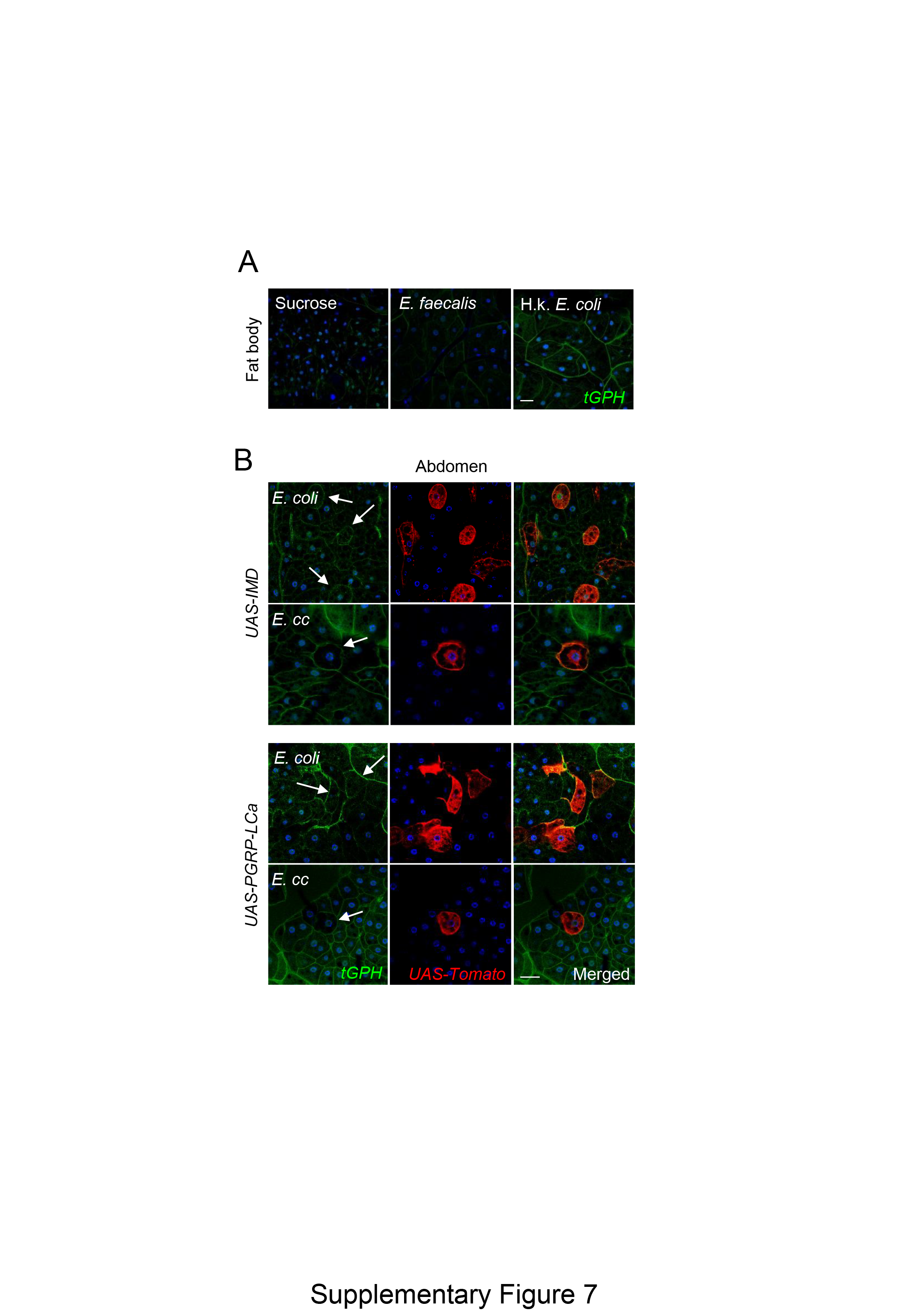

Supplement: S7 Fig — Confocal images of fat body from adult flies fed 2 days with either sucrose, or a mixture of sucrose + E. faecalis or heat killed (H.k) E. coli, and showing the tGPH marker (green). Ingestion of heat killed E. coli promote recruitment of tGPH at the cell surface of adipocytes, compared to a sucrose diet. (B) Confocal images of fat body showing clones of adipocytes overexpressing IMD or PGRP-LCa (red) and the tGPH marker (green), from flies fed 1 day with E. cc or 2 days with E. coli. Fat body clones over expressing either IMD or PGRP-LCa do not affect tGPH recruitment at the cell surface of adipocytes. Flies of the following genotypes were used: w1118/w1118; tGPH/tGPH (A) and w1118, CoinFLPGal4/w1118; tGPH, UAS-CD4::Tomato/hs-FLP.G5, TubGal80ts; UAS-IMD or UAS-PGRP-LCa/+. Scale bar is 20 μm. (TIFF) [file pgen.1010098.s007.tiff]
