# Supplementary material for: Gut-derived peptidoglycan remotely inhibits bacteria dependent activation of SREBP by Drosophila adipocytes
Source: PLoS Genet. 2022 Mar 4;18(3):e1010098. doi: 10.1371/journal.pgen.1010098 (PMC8926189; doi:10.1371/journal.pgen.1010098)
Supplement: Attachment — Submitted filename: Response to reviewers Charroux and Royet.docx [file pgen.1010098.s009.docx]

**Dear reviewers,**

Thank you for your comments and suggestions, some of which help us to improve the manuscript as shown below.

Bernard Charroux and Julien Royet

**Reviewer #1:**

In this study, Charroux and Royet investigated the molecular mechanism by which gut bacteria modulate lipid momeostasis via cross-regulation between Insulin-SREBP signaling and PGN-IMD signaling. They first observed that specific gut bacterial species including E. coli and Erwinia carotovora carotovora (ECC) could activate an SREBP-dependent lipogenesis in enterocyte, fat body and ovaries. They next showed that insulin singaling activation is requried for bacterial-induced SREBP activation. Interestingly, bacterial-induced SREBP activation is abolished under PGRP-LB mutant backgroud, which could be reverted by the simultanous IMD pathway inactivation, demonstrating that IMD pathway activation is able to antagonize bacterial-induced SREBP activation. Clonal analysis further confirmed that bacterial-induced SREBP activation in fat body cells is repressed by forced activation of IMD pathway in a cell –autonomous manner. They observed that PGRP-LB mutant animals showed reduced lifespan upon bacterial infection. Importantly, tissue-specific inactivation of IMD pathway in bacterial-infected PGRP-LB mutant animals revealed that inactivation of IMD pathway in fat body, but not muscle or enterocytes, is sufficient to improve the surivival of PGRP-LB mutant animals. Taken together, they concluded that cross-regulation between IMD-NF-kappaB pathway and Insulin-FOXO and SREBP pathway is required for lipid homeostasis and host survival during enteric infection, highlighting the importance of a balance between metabolic and immune signaling.

General comments.

Immune-metabolic cross-talk is an important issue, and plays a pivotal role in physiological adaptation at an organismal level during inflammation. However, at present, the precise mechanism governing this cross-talk is poorly understood. In this context, I found that this study is novel and important in the field. They used an elegant genetic approach to clearly demonstrate their hypothesis and many of the experiments are meticulously controlled. I have only few comments and suggestions that would improve the significances of the present work.

**Major Point**

Perhaps, the metabolic changes during the course of infection “might” be dynamic. For example, in the early phase of infection (e.g. within 30 min to few hr), the insulin signaling may be somewhat inactivated or down-regulated (possibly by infection-induced NF-kappaB pathway activation) and lipogenesis is blocked. This process may be required for allocating energy for the immune response and/or for the inhibition of excess ROS production. For example, a recent paper (Immunity, 2020, 52, 374pp) showed that septic infection rapidly induces the excretion of hemolymph lipid to prevent ROS production. They showed that lipid level started to be depleted 1 hr after infection, and remained at a very low level over 6 hr (less than 10% compared with the level of control animal!), and then started to be replenished (that “may” certainly require lipogenesis in adipocytes) and reached to normal level at 24hr. They found a similar decrease of lipids after oral bacterial infection with ECC (see figure S1B of Immunity, 2020). Therefore, it may be possible that animal uses or excretes lipids while preventing lipogenesis, at least in an early time point of infection. However, in the late phase of infection (~12 hr or later), animals may actively operate insulin signaling to promote lipogenesis for the lipid homeostasis (e.g. in part to replenish the hemolymph lipid). In this context of scenario, it is interesting to discuss these important points in the discussion section or to examine time-course activation of insulin may be signaling because the authors examined insulin activation (SREBP activation) mainly in the late phase of infection (~ few days following infection).

We agree with the reviewer that timing and dynamics are important parameters to consider when one analyzes the interplay between immune activation and metabolism. Since the molecular dialogs that govern such processes can be very rapid, with different timescale and could involve many feedbacks, it is very difficult to pinpoint the good timepoint to look at. As proposed by the reviewer, we have added a paragraph in the discussion to mention this aspect and to discuss our results in the context of the Immunity 2020 paper. We also would like to emphasize that dead bacteria are also able to induce fat body SREBP (we added these data and discussed them) suggesting that SREBP activation is probably not secondary to the infectious process by itself but more by a direct effect of a bacteria-derived compound, such as aa.

1. Why is “adipocyte-specific” lipogenesis (SREBP activation) important for host survival following gut infection? It might be interesting to discuss this issue in the discussion section.

We have added a part in the discussion session to propose this hypothesis in relation to the Immunity 2020 paper.

2. Is forced insulin signaling activation (e.g. InR overexpression or FOXO inactivation) in the fat body of PGRP-LB mutants sufficient to confer host resistance to bacterial infection?

As proposed by the reviewer, we have tested whether forced activation of the insulin pathway in the fat body of *PGRP-LB* mutants (using the Gal4-UAS system) would be sufficient to, at least partially, rescue the sensitivity of *PGRP-LB* mutants to *Ecc* oral bacterial infection. We have done the experiences via three means. (1) Overexpression of the wild-type Insulin Rc in the fat body of *PGRP-LB* mutants flies. (2) Overexpression of a constitutively active form of the Insulin Rc (InR**^act^**) in the fat body of *PGRP-LB* mutants flies. (3) RNAi-mediated inactivation of Foxo, a negative transducer of the insulin pathway, in the fat body of *PGRP-LB* mutants flies. The results obtained, shown below, gave contradictory results. While overexpression of the wild-type InR triggers a slight but significant rescue of the *PGRP-LB* mutant phenotype, no effects were observed with InR^act^ overexpression and an exacerbation of the phenotype was noticed following inactivation of Foxo in the fat body. Although we believe that the InR experiment is the more “physiological’ of the three because 1) the other two trigger a constitute activation of the pathway while the one with the InR is expected to “only” sensitize the system, 2) Foxo implication is not restricted to the Insulin pathway, we decided not to include them in the manuscript. It is indeed very difficult to interpret phenotypes triggered by a prolonged overexpression or inactivation of key metabolic signaling players.

**Figure legend:** Survival analysis of *PGRP-LB^Δ^* mutant flies orally infected with *E. cc*. The expression of *UAS-InR^WT^* (BL#8262) or *UAS-InR^act^* (BL#8248) or *UAS-dFoxo^IR^* (VDRC#v106097) using the fat body Gal4 driver *r4^Gal4^* have distinct effects of *PGRP-LB^Δ^* mutation on fly’s survival. Flies overexpressing *UAS-InR^WT^* or *UAS-dFoxo^IR^* have respectively, a significantly extended and reduced lifespan compared to the corresponding control flies (Log-rank (Mantel-Cox) test, *p < 0.05). Flies of the following genotypes were used: *w^1118^*/*w^1118^; PGRP-LB^Δ^, r4^Gal4^* /*PGRP-LB^Δ^* or *w^1118^*/*w^1118^; UAS-InR^wt^* or *UAS-InR^act^* or *UAS-dFoxo^IR^/+; PGRP-LB^Δ^*/*PGRP-LB^Δ^* or *w^1118^*/*w^1118^; UAS-InR^wt^* or *UAS-InR^act^* or *UAS-dFoxo^IR^/+; PGRP-LB^Δ^, r4^Gal4^* /*PGRP-LB^Δ^*. n=3 indicates that three independent experiments were performed.

**Reviewer #2:**

In this ms, Charroux and Royet describe a systemic circuitry that allows metabolic adaptation in response to oral bacterial infection in Drosophila. They propose that, upon infection, SREBP activation and lipid droplets are induced in adipocytes, by coordinating insulin signaling induction with blocking, via PGRP-LB-dependent inactivation of bacterial PGN, of IMD-dependent SREBP inhibition. PGRP-LB loss antagonizes insulin-dependent SREBP activation, leading to chronic inflammation and premature fly death. Overall, the findings provide interesting insights in adipocytes immune-metabolic regulation, are clearly presented and experiments are well conducted. However, I do have some concerns that should be addressed to support the conclusions.

**Major comments:**

- the authors’ model is consistent with a physiological response to infection that favors anabolism vs catabolism in adipocytes. How this response is induced should be experimentally clarified. For example, if the authors are proposing that this response is triggered, as suggested by their model, to balance protein catabolism induced in the gut by bacterial infection, this idea should be experimentally tested in their settings. One possibility would be that blocking bacteria-induced protein catabolism in the gut prevents SREBP and lipid droplets induction in adipocytes.

We agree that it is an important hypothesis to consider. As proposed by this reviewer, we have experimentally tested whether blocking protein catabolism in the gut by inhibiting the autophagy protein ATG1, will prevent SREBP activation in fat body of *E. coli* orally infected flies. The results of this experiment showed that protein catabolism in the gut is indeed required for SREBP activation in fat body. When ATG1 is downregulated in enterocytes, *E. coli* is no longer able to remotely activate SREBP in adipocytes. These new results which nicely clarify the point raised by this reviewer is now presented in Figure 3I.

Furthermore, the implied requirement of DILPs for insulin signaling activation in adipocytes should be experimentally assessed in the authors’ settings.

We agree with the reviewer that this experiment will strengthen the manuscript. We have tested it by overexpressing a dominant negative version of the InR (InR^DN^) in the fat body only. The data obtained demonstrate that overexpression of InR^DN^ in the fat body completely prevents SREBP activation in *E. coli* orally infected flies. These new results, now presented in Figure 3G demonstrated that the insulin Rc is tissue-autonomously required in the fat body to allow gut bacteria-dependent SREBP activation.

It would also be important to more generally assess whether catabolic pathways are inhibited in adipocytes of infected flies, for example those dependent on the activity of AMPK, which indeed was suggested to inhibit SREBP in Drosophila adipocytes (Bertolio et al. Nat Comm 2019).

As mentioned by the reviewer, previous work has shown that activation of AMP-activated protein kinase (AMPK) by ECM stiffening inhibits SREBP1 activation. We could therefore expect that SREBP activation in the fat body is secondary to an inhibition of AMPK. To test this hypothesis, we analyzed the effect of a constitutively active form of AMPK (AMPK^act^) on SREBP induction in fat body of *E. coli* infected flies. If bacteria-dependent SREBP activation depends on AMPK downregulation, it should not happen in cells expressing a constitutive form of AMPK. Our new results demonstrate that clones of fat body that express AMPK^act^ are as competent as wild-type neighboring cells to activate SREBP. These new results, which suggest that SREBP is activated independently of AMPK, are presented below. They were not included in the text since they are negative results.

**Figure legend:** Confocal images of fat body showing clones of adipocytes overexpressing an activated form of AMPKα (*UAS-AMPKa^T184D^*, BL#32110) (red) and activation of *LexA::SREBP* (green), from flies fed 2 days with *E. coli*. Fat body clones over expressing do not affect *LexA::SREBP* activation. Flies of the following genotypes were used: *w^1118^*, *CoinFLP^Gal4^*/*w^1118^*; *LexA::SREBP, LexAop-CD8-GFP-2A-CD8-GFP, UAS-CD4::Tomato/UAS-AMPKa^T184D^; hs-FLP.G5, Tub^Gal80ts^/+.* Scale bar is 20 μm.

- in bacterial-infected PGRP-LB mutants, the requirement of PGRP-LC and other IMD pathway components (tak1, rel, ikk) for inhibition of SREBP activation, beside Dredd and dFadd, should be assessed.

As required by the reviewer, we have extended the epistatic analysis to two other key actors of the IMD/NF-kB pathway. The membrane-associated peptidoglycan receptor PGRP-LC and the transactivator Relish. In both mutant backgrounds, the SREBP inhibition observed in orally infected *PGRP-LB* mutants is no longer present. These new data, which nicely reinforced our model, and demonstrate that the upstream PGN sensing mechanism and the downstream transcriptional response are also required to downregulate fat body SREBP activation, are now presented in Supplementary Fig. 4

It is unclear why different dredd alleles where used in figs5/6. Both should be tested in each.

According to the data presented in the original Dredd paper (Leulier et al, EMBO reports 2000), both alleles act as very strong Dredd allele. They both prevent Dipt activation in fat body of infected adult flies, similarly to the (D3) Dredd Deficiency. We used different alleles for experimental reasons. However, as shown below, the fat body of *Dredd^F64^* mutant flies orally infected with *E. coli* looks like the one from *Dredd^D55^* mutant flies. This results together with those showing that *PGRP-LC, Fadd, Dredd and Relish* are required to block fat body SREBP activation upon *E. coli* infection let us believe that the IMD pathway is indeed implicated in SREBP inhibition.

**Figure legend:** Confocal images of fat body from flies fed 8 days with sucrose showing lipid droplets labelled with Bodipy (green). The presence of large lipid droplets characteristic of the loss of function allele *Dredd^D55^*is also observed for the *Dredd^F64^* allele.

Related to this, rescue of fly survival by dFadd RNAi in fig6D is mild compared to fig6B. Is this due to pathway redundancy? What is the efficiency of dFadd RNAi?

There is different hypothesis. Pathway redundancy is one of them. But we rather favor the fact that inactivating a gene via RNAi is not as efficient as inactivating a gene by mutation which can, in certain cases, generate complete loss-of-function alleles. As mentioned above, the *Dredd^D55^* allele used in Fig. 6b is indeed considered to be a null allele (Leulier et al, EMBO reports 2000). It is also unclear how ubiquitous and uniformly expressed are the Gal4 drivers and, in our case, DaGal4 (although it is always described as pan tissues). This kind of limitation exist for every single experiment using the Gal4-UAS system. The Fadd RNAi is efficient in silencing the IMD pathway and has been used in many studies to downregulate the IMD pathway. As shown below, we have compared, via qRT-PCR, IMD pathway activation levels (using diptericin mRNA level as a proxy), in *Dredd* mutant and flies that overexpress a Fadd RNAi transgene. The results confirm that the Fadd RNAi is rather efficient in inhibiting the IMD pathway but not as efficient as a null allele of one the core component such as Dredd.

**Figure legend:** Histograms showing the expression of *Diptericin* measured by q-RT-PCR and performed with mRNA extracted from adult abdominal carcasses of adult flies fed 2 days with either sucrose, on with a mixture of sucrose + bacteria (*E. cc,* OD_600_=100). The mRNA level in non-infected control flies was set to 1 and values obtained with indicated genotypes were expressed as a fold of this value. Histograms correspond to the mean value ± SD of three experiments. Flies of the following genotypes were used: *w^1118^/w^1118^* (Control) or *w^1118^/w^1118^;; da^Gal4^* /+ or *w^1118^/w^1118^;; da^Gal4^* /*UAS-dFadd^IR^* or *w^1118^, Dredd^D55^/w^1118^, Dredd^D55^*.

- in most experiments, SREBP activation is assessed just with the LexA::SREBP reporter activation. Why the pattern of LexA::SREBP reporter activation (fig2a,b) in ovaries is different from that of Gal4::SREBP reported Kunte et al (ref 22)?

This is difficult to say. One obvious reason could be that the raising conditions are not exactly the same. One the most probable explanation is that, since the fly food composition is not the same in both studies, this can impact the expression pattern. Genetic background would be another possibility.

LexA::SREBP reporter activation in key experiments should be paralleled by immunofluorescence to detect nuclear localization of endogenous SREBP and induction of SREBP targets

We are not sure why using the Gal4::SREBP and LexA::SREBP reporter will not be good assay to monitor SREBP activation. This has been used in many studies. In contrast, immuno-fluorescence with an anti-SREBP has rarely been performed in fly tissues. In addition, the commercially antibody used in Bertolio et al. Nat Comm 2019 (anti-SREBP1 H160; Santa Cruz Biotechnology sc8984) is no longer for sale. We have however ordered and tested another antibody against SREBP. Our new data show that while *E.cc* feeding induces a weak nuclear translocation of SREBP not observed in control conditions, *E. coli* feeding triggers a stronger SREBP nuclear staining. These data, which are totally coherent with those obtained with the LexA and Gal4 reporters, and hence reinforced our model, are presented Fig. 1C.

Relate to this, the effect of PGRP-LB mutation reducing SREBP activation, as quantified with ACS induction in fig 1C, is unclear. Is the difference in ACS induction between control and PGRP-LB mutant statistically significant?

No, it is not. This is a tendency.

Also, lipid droplets in fat bodies should be quantified

As requested by the reviewer, the quantification has been done and is now shown.

**Minor issues:**

- all figure panels should include controls, whose lack often obliges the reader to pick them in other figures (just as examples, fig5a, NF-kB activation with PGRP-LCa or IMD overexpression

We have added the requested controls.

lifespan of non-infected flies in fig6B

We have added life span of non-infected flies (Figure S6)

- all figure panels should be referenced to in the text (for example fig. S4d)

This is been corrected.

Reviewer #3, R0

Mainly, the authors are confounding microbiome and a gut pathogen, which is not correct and confuses interpretation. In addition, the authors would need to either provide more support (genetic evidence) or temper some of the conclusions.

We do not understand the reviewer’s comments as we are not using the term microbiome in the text. As for microbiota, it is used only once in the introduction to cite previous work. We clearly state that we are studying the consequences of an enteric bacterial infection.

The text also needs some copy editing (I think for gut born they mean gut-derived, or at least the latter term would be more appropriate). Below are more detailed comments:

We have change gut-born into gut-derived although we believe that the PGN is released by bacteria growing in the gut lumen. So, we can probably say that it is born there.

The present manuscript by Charroux and Royet attempts to demonstrate activation of the lipogenic protein, Sterol Regulatory Element Binding Protein (SREBP), by enteric infection with E. coli or Erwinia carotovora carotovora. Convincingly the authors demonstrate increase transcription of SREBP by enteric infection with Ec or Ecc in the fat body and enterocytes of Drosophila. IMD/NF-kB signaling antagonizes the expression of SREBP. The manuscript struggles from several weaknesses and requires additional attention prior to publication.

The justification for the generation of a second SREBP reporter construct is not clear, so what benefits this construct confers is also unclear

The rationale beyond the generation of this new reporter (LexA-SREBP) is that, without it, we would not be able to use the Gal4-UAS system (flip out clones and overexpression experiment using Gal4). We would have been happy to rely only on the Gal4 reporter but we had no other choice to further dissect the molecular mechanisms involved.

The authors frequently switch between the use of the GAL4::SREBP reporter and the LexA::SREBP reporter without proper justification for this switch. Comparative expression analysis was not performed so the ability of the two reporters to function equivalently is unclear

We do not understand the reviewer comment since the Supplementary Fig 2 is fully dedicated to this issue. The expression patterns of LexA::SREBP and Gal4::SREBP are very similar, indeed.

The molecular details of the reporter are, per the methods, “available upon request,” but these details must be published in a final version of the manuscript

We have provided the requested information in the material and methods section. Without going into details, the LexA construct is exactly the same as the gal4 version. Exact same protocol (same restriction sites and oligos) as the one described in (Kunte et al 2006) except that we cloned the lexA-relA ORF cDNA instead of Gal4 cDNA. The information is now provided in the Material and Method section.

The LexA reporter construct consists of a LexA-RelA fusion protein thus taking advantage of the RelishA transactivation domain to drive the expression of the reporter. Per the authors own data, NF-kB signaling (which is promoted by RelishA) antagonizes the production of SREBP. It is unclear if the RelA transactivation domain is sufficient to drive the expression of NF-kB effectors and thus the authors, by virtue of using their LexA reporter, may be introducing artifacts into their own data. Per the information presented, this reporter is likely inappropriate and confounds all experiments using the LexA promoter. This should be urgently addressed.

First, we would like to mention that only the activation domain of RelA is used here. Second, we would like to emphasize that the results obtained are the same with the LexA and the Gal4 reporters and that the latter has nothing to do with NF-kB. We have nonetheless performed experiments to see whether the LexA containing construct is interfering with NF-kB signaling. These experiments, whose results are shown below, demonstrate that flies carrying LexA::SREBP construct behave as control flies as far as NF-kB dependent AMP production is concerned. The LexA::SREBP is not interfering with NF-KB activation.

**Figure legend:** Histograms showing the expression of *Diptericin* measured by q-RT-PCR and performed with mRNA extracted from adult abdominal carcasses of adult flies not infected or infected by septic injury with a needle dipped into a solution of *E. coli* (OD_600_=50). Before sceptic injury, adult flies were raised 48h on the indicated media in order to activate (with *E.coli*), or not (with sucrose and regular food), the processing of both the *Gal4::SREBP* or the *LexA::SREBP* fusion proteins. The mRNA level in non-infected control *Gal4::SREBP* flies was set to 1 and values obtained with indicated genotypes were expressed as a fold of this value. Histograms correspond to the mean value ± SD of three experiments.

Antibiotic clearance is used by the authors to generate axenic flies prior to enteric infection with Ec or Ecc. Per the methods flies are flipped off of food with antibiotics for 24 hours prior to infection; however, whether the food is sterile in the absence of antibiotics is unclear. Thus, within those 24 hours the flies may begin intaking microbes from the food/environment that confound the attribution of results to only Ec or Ecc.

In our conditions, flies that have been kept for 24 h and antibiotics and then on regular food are fully axenic. These data are shown below.

**Figure legend:** Internal bacterial load of germ-free Drosophila individuals, either control or *PGRP-LB^Δ^* mutants, raised in different media or after monoassociation with the indicated bacteria. Histograms correspond to the mean
value +/- SD of 15 experiments. ns: not significantly different. Flies of the following genotype were used: *w^1118^*/*w^1118^* (Control) or *w^1118^*/*w^1118^; PGRP-LB^Δ^,* /*PGRP-LB^Δ^* (*PGRP-LB^Δ^*). 15 individuals were analyzed separately for each condition and for each genotype. The data set used for this histogram is shown below.

| Control | Control | Control | Control | Control | *PGRP-LB^Δ^* | *PGRP-LB^Δ^* | *PGRP-LB^Δ^* | *PGRP-LB^Δ^* | *PGRP-LB^Δ^* |
| --- | --- | --- | --- | --- | --- | --- | --- | --- | --- |
| ATB | Regular food | Sucrose | *E. coli* | *E. cc* | ATB | Regular food | Sucrose | *E. coli* | *E. cc* |
| 0 | 0 | 0 | 35200 | 35200 | 0 | 0 | 0 | 45800 | 23500 |
| 0 | 0 | 0 | 25200 | 22200 | 0 | 0 | 0 | 32300 | 35000 |
| 0 | 0 | 5 | 41200 | 33300 | 0 | 0 | 0 | 45600 | 46300 |
| 0 | 0 | 0 | 35800 | 24200 | 0 | 0 | 1 | 23600 | 34800 |
| 0 | 0 | 0 | 28500 | 28200 | 0 | 1 | 0 | 21900 | 21800 |
| 0 | 0 | 1 | 31500 | 26300 | 0 | 0 | 0 | 36600 | 36900 |
| 0 | 0 | 0 | 42800 | 31200 | 0 | 0 | 0 | 32200 | 20200 |
| 0 | 0 | 0 | 24800 | 45200 | 0 | 0 | 0 | 29600 | 42500 |
| 0 | 0 | 0 | 25600 | 47100 | 0 | 0 | 0 | 24400 | 28900 |
| 0 | 0 | 0 | 33500 | 49600 | 0 | 0 | 0 | 25300 | 38700 |
| 0 | 0 | 0 | 29800 | 30200 | 0 | 0 | 0 | 22800 | 39700 |
| 0 | 0 | 0 | 36500 | 50000 | 0 | 0 | 2 | 31600 | 21900 |
| 0 | 0 | 2 | 33500 | 27600 | 0 | 0 | 0 | 28500 | 48200 |
| 0 | 1 | 0 | 39800 | 37200 | 0 | 0 | 0 | 37200 | 26500 |
| 0 | 0 | 0 | 48200 | 25900 | 0 | 0 | 0 | 45600 | 20300 |
|  |  |  |  |  |  |  |  |  |  |

**Method:** Bacterial load of surface-sterilized individuals was quantified by plating serial dilutions of lysates obtained from 1 individual on nutrient agar plates (LB plates supplemented with rifampicin [100 μg/ml] for Ecc). Homogenization of individuals was performed using the Precellys 24-tissue homogenizer (Bertin Technologies, France) and 0.75/1 mm glass beads in 800 μl of the appropriate bacterial culture medium. Bacterial loads were analyzed 48 hrs post monoassociation of adult’s flies.

Per the methods, infections use microbe contaminated food. The methods make no mention of how a “sucrose only” infection is accomplished. Throughout the narrative the authors confusingly say sucrose only and regular food separately which makes it unclear if the flies were fed food supplemented with sucrose or some alternative method. This should be clarified. If sucrose only and regular food are two distinct groups then all analyses across the figures should be redone to include the regular food groups.

Regular food and sucrose are indeed two different groups. However, to clarify the text we have decide to remove data related to regular food. Our point here is to compare the flies fed on sucrose only or on sucrose and different bacterial species.

On an almost per-figure basis the authors exclude either Ec or Ecc from their results though a clear justification for this is not made. Without having both models in every experiment the results cannot be extended to enteric infection by both Ec and Ecc.

We use both species in most experiments. However, one point of the manuscript deals with the reduced SREBP activation following *E. cc* infection and the reason(s) for it. We hypothesize that PGN-dependent IMD pathway activation in the fat body (seen with *E. cc* but not with *E. coli*) is responsible for it. When we test this hypothesis, we, logically, only use *E.cc.* On the other hand, *E. coli* is inducing a stronger SREBP signal. When we want to dissect the pathway upstream of SREBP activation, we use *E. coli.*

Figure 2 includes mated vs virgin analysis for other organs such as the fat body, but this type of analysis is notably absent for the ovaries. The ovaries should be analyzed in virgin and mated conditions

This has been done and included in the new figures.

Figure 2D is poorly controlled. The male midgut data does not include uninfected flies. The female midgut data does not include a mated sucrose group or a virgin infected group. Appropriate controls should be included.

This has been added. We do not use *E.cc* in this figure since the goal of the experiments presented is to identify the conditions required for bacterial dependent SREBP activation. Since *E.cc* is a poor inducer (due to its simultaneous ability to activate the IMD pathway) there is no point to use it. We only use *E. coli* which give the strongest signal.

The authors should provide more context for the usage of tGPH as a reporter of insulin

We have modified the text to do so.

Figure 3A shows what could be interpreted as similar levels of tGPH expression between regular food and the infection groups. Yet, the qPCR analysis of Thor in 3B does not include the regular food group. Per the results shown in 3A one could reasonably attribute the activation of insulin signaling as being promoted by the food instead of the infectious microbes*.* Thor expression should be analyzed in the regular food group.

This has been done. Data are presented in Fig. 3b.

Lines 196-197 say “PGN-dependent IMD/NFkB pathway activation” but this is not necessarily the case. PGN-induced should be used so as to not imply dependency on PGN as opposed to the IMD signal.

Our new data shown Supplementary Fig. 5 show that, in addition to core intracytoplasmic compounds such as *Fadd* or *Dredd*, *PGRP-LC* is also required in order for *E. cc* to dampen SREBP activation in fat body. This strongly suggests that the effects are indeed mediated by a PGN-dependent IMD signaling activation*.*

Lines 219-220 comment on the ability of PGRP-LB in combating chronic infection by dampening NF-kB signaling and increasing lipogenicity. Bacterial loads were not analyzed in this study and thus this claim seems unsubstantiated.

We have adapted the text.

The discussion section is unclear in some areas, so a more careful revision of the discussion section should be done

The discussion has been modified to respond to the reviewer comment

Samples sizes are not included in the tables or in the methods. This information should be included for the interpretation of significance.

They have been added.
